# PDGFRA in vascular adventitial MSCs promotes neointima formation in arteriovenous fistula in chronic kidney disease

**DOI:** 10.1172/jci.insight.137298

**Published:** 2020-11-05

**Authors:** Ke Song, Ying Qing, Qunying Guo, Eric K. Peden, Changyi Chen, William E. Mitch, Luan Truong, Jizhong Cheng

**Affiliations:** 1 Department of Stomatology, Tongji Hospital, Tongji Medical College, Huazhong University of Science and Technology, Wuhan, China.; 2 Selzman Institute for Kidney Health, Section of Nephrology, Department of Medicine, Baylor College of Medicine, Houston, Texas, USA.; 3 Department of Vascular Surgery, DeBakey Heart and Vascular Institute, Houston Methodist Hospital, Houston, Texas, USA.; 4 Michael E. DeBakey Department of Surgery, Baylor College of Medicine, Houston, Texas, USA.; 5 Department of Pathology and Genomic Medicine, Houston Methodist Hospital, Houston, Texas, USA.

**Keywords:** Nephrology, Stem cells, Chronic kidney disease, Mouse stem cells, Signal transduction

## Abstract

Chronic kidney disease (CKD) induces the failure of arteriovenous fistulas (AVFs) and promotes the differentiation of vascular adventitial GLI1-positive mesenchymal stem cells (GMCs). However, the roles of GMCs in forming neointima in AVFs remain unknown. GMCs isolated from CKD mice showed increased potential capacity of differentiation into myofibroblast-like cells. Increased activation of expression of PDGFRA and hedgehog (HH) signaling were detected in adventitial cells of AVFs from patients with end-stage kidney disease and CKD mice. PDGFRA was translocated and accumulated in early endosome when sonic hedgehog was overexpressed. In endosome, PDGFRA-mediated activation of TGFB1/SMAD signaling promoted the differentiation of GMCs into myofibroblasts, extracellular matrix deposition, and vascular fibrosis. These responses resulted in neointima formation and AVF failure. KO of *Pdgfra* or inhibition of HH signaling in GMCs suppressed the differentiation of GMCs into myofibroblasts. In vivo, specific KO of *Pdgfra* inhibited GMC activation and vascular fibrosis, resulting in suppression of neointima formation and improvement of AVF patency despite CKD. Our findings could yield strategies for maintaining AVF functions.

## Abstract 2

Hedgehog signaling regulates PDGFRA in vascular adventitial mesenchymal stem cells to promote neointima formation in arteriovenous fistula in chronic kidney disease.

## Graphical abstract

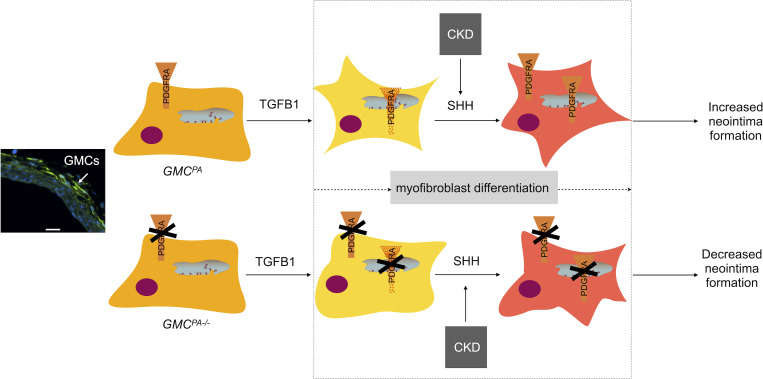

## Introduction

A major problem for patients with chronic kidney disease (CKD) is that their clinical course progressively develops into end-stage kidney disease (ESRD), thereby having a higher risk of mortality due to cardiovascular disease (1). For patients treated with hemodialysis, it is recommended that an arteriovenous fistula (AVF) be created because outcomes of AVF functions provide a superior dialysis access when compared with results of arteriovenous grafts or catheters. Unfortunately, the 2-year maturation failure rate of AVFs is approximately 50% (2).

A major cause for AVF failure is the development of a neointima at the venous anastomosis (3). Neointima cells are derived from multiple sources. Adventitial cells can migrate through the arterial media to form neointima (4, 5). The progenitor (i.e., stem) cells in the adventitia of artery and vein can be activated and transdifferentiated into neointima cells after vessel injury (6–9). However, the contribution of adventitial stem cells to AVF fibrosis and neointima formation has not been deciphered.

PDGFR subunit A ( PDGFRA) acts as a receptor in perivascular cells and is believed to play a major role in the regulation of blood vessel development. PDGFRA is expressed in mesenchymal stem cells (MSCs) and regulates their differentiation (10). When PDGFRA is activated, it leads to the accumulation of myofibroblasts and matrix deposition that subsequently develop into tissue fibrosis (10–12).

Hedgehog (HH) signaling pathway regulates neovascularization during the development of blood vessels (13). The components of the sonic HH (Shh) signaling molecule are found in the adventitia of normal adult blood vessels (14). When Shh binds to patched 1 (Ptch1) receptor, it antagonizes the repressor function of the smoothened (Smo) gene, which leads to Smo phosphorylation and activation of GLI1/2 transcription factors (15). GLI1-positive MSCs can differentiate into vascular smooth muscle cells (VSMCs) after vascular injury and during vascular calcification (16, 17).

We aimed to identify whether GMCs of the adventitia contribute to neointima formation in AVFs placed in CKD mice. We determined the function of PDGFRA in regulating GMC transdifferentiation, vascular fibrosis, and CKD-induced neointima formation. Our results uncover therapeutic targets for preventing formation of neointima in AVFs.

## Results

### Increased expression of PDGFRA was associated with activated HH signaling pathway in AVFs from patients with ESRD.

**Figure 1:**
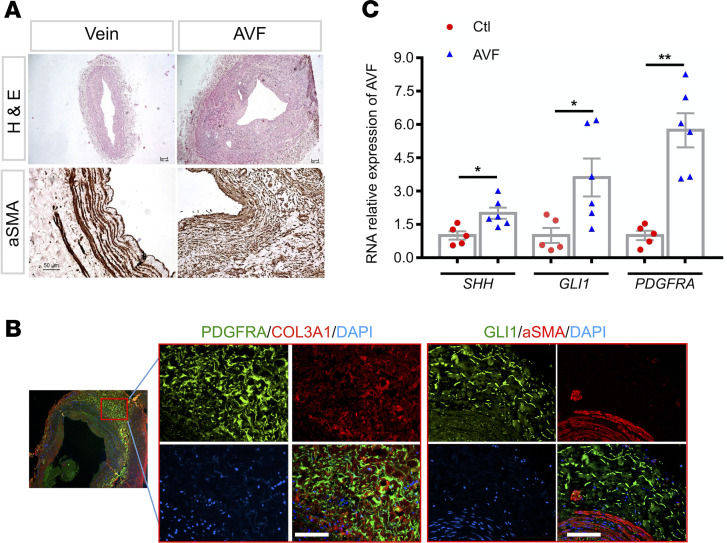
Increased expression of PDGFRA and activation of HH signaling were observed in vascular adventitial MSCs from patient AVFs. (**A**) A smaller lumen and a thicker wall of AVFs in patients with ESRD were detected by H&E staining. α-SMA–positive cells were accumulated in the neointima areas. Scale bars: 100 μm and 50 μm. (**B**) Immunofluorescence staining revealed that the abundance of PDGFRA-positive cells were specifically located in adventitia and colocalized with COL3A1 in AVFs from patients with ESRD. Meanwhile, plenty of GLI1-positive cells were also detected in these areas. Scale bars: 100 μm. (**C**) Total RNAs were isolated from these human AVFs for qRT-PCR analysis. Increased expression of *PDGFRA*, *SHH*, and *GLI1* were observed (*n* = 5 for Ctl vein samples, *n* = 6 for AVF samples). The levels of mRNA expression are presented as folds of Ctl group after normalization to β-actin. *P < 0.05, **P < 0.01 versus Ctls (*t* test). HH, hedgehog; MSCs, mesenchymal stem cells; AVFs, arteriovenous fistula; ESRD, end-stage kidney disease; α-SMA, α-smooth muscle actin; COL3A1, collagen type III α 1; Ctl, control.

A smaller lumen and a thicker wall of AVFs were detected in human specimens from patients with ESRD. α-Smooth muscle actin–positive (α-SMA/ACTA2) cells were accumulated in the neointima (Figure 1A). Immunofluorescence staining revealed that the abundance of PDGFRA-positive cells were specifically located in AVF adventitia and colocalized with collagen type III α 1 (COL3A1) in AVFs from patients with ESRD. Plenty of GLI1-positive cells were also detected in AVF adventitia (Figure 1B). Furthermore, marked increases of mRNA levels of *PDGFRA*, *SHH*, and *GLI1* were detected in AVFs from patients with ESRD (Figure 1C). These results indicate that increased expression of PDGFRA in AVF adventitia was associated with activation of HH signaling pathway.

### CKD activated HH signaling and increased the expression of PDGFRA in mice vascular adventitia.

To determine if CKD regulates HH activity, we studied *Gli1^lz+/–^* reporter mice, in which a “knockin” of β-galactosidase (*LacZ*) is inserted into the first coding exon of GLI1, replacing exons 2–7 (that encode the entire N-terminal and zinc-finger domains of GLI1). X-gal staining, the presence of a blue-colored product (Supplemental Figure 1A; supplemental material available online with this article; https://doi.org/10.1172/jci.insight.137298DS1), was used to reflect the activation of β-galactosidase (18). In *Gli1^lz+/–^* mice, the expression of LacZ was increased in reflection of the activation of HH signaling (because GLI1 itself is a target of HH). Specifically, GLI1-positive signals were restricted to the adventitial layers of arteries and veins (Supplemental Figure 1B).

**Figure 2:**
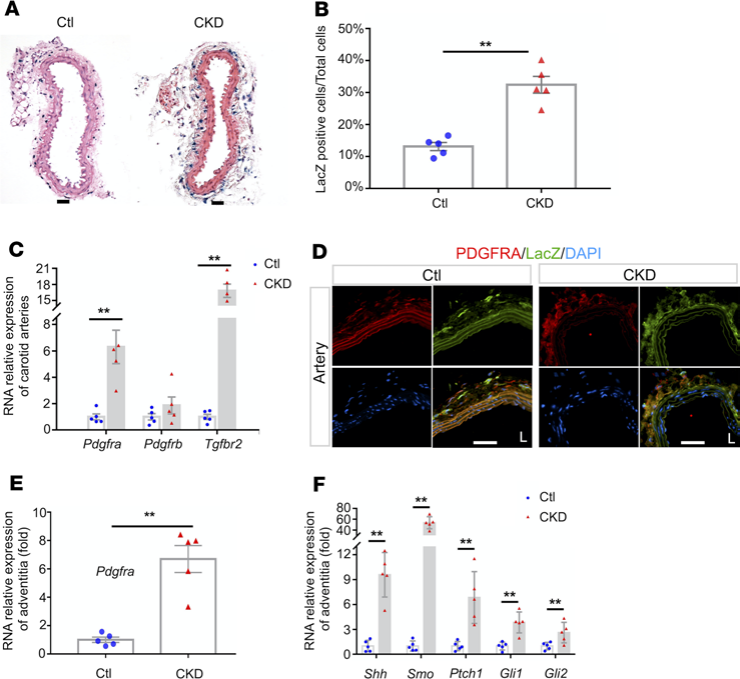
CKD stimulates the expression of PDGFRA and activation of HH signaling in vascular adventitial MSCs in mouse vessels. (**A** and **B**) Representative images of the arteries from Ctl and CKD mice that were isolated from *Gli1^lz+/–^* reporter mice. Scale bars: 100 μm. (**B**) The percentage of the X-gal–positive cells was calculated. *n* = 5, **P < 0.01 versus Ctls (*t* test). (**C**) The mRNA levels of several cellular receptors are markedly induced in carotid arteries from mice with CKD (*n* = 5). The levels of mRNA expression are presented as folds of Ctl group after normalization to β-actin. **P < 0.01 versus Ctls (*t* test). (**D**) Representative images show the immunofluorescent staining of PDGFRA and GLI1 (LacZ) in adventitial cells of the common carotid artery in Ctl mice and in mice with CKD that were created in *Gli1^lz+/–^* reporter mice. Scale bars: 50 μm. (**E** and **F**) Adventitia layers were isolated from thoracic aortas of Ctl and CKD mice, and total RNAs were extracted from these tissues. The expression of PDGFRA (**E**) and HH signaling molecules (**F**) were determined by RT-qPCR (*n* = 5). The levels of mRNA expression are presented as folds of Ctl group after normalization to β-actin. **P < 0.01 versus Ctls (*t* test). CKD, chronic kidney disease; HH, hedgehog; MSCs, mesenchymal stem cells; Ctl, control.

In mice with advanced CKD, there were increased LacZ-positive cells in the adventitia of the carotid artery in *Gli1^lz+/–^* reporter mice versus the results obtained in control (Ctl) mice (Figure 2, A and B). We found that the mRNA levels of several receptor tyrosine kinases (e.g., *Pdgfra*, *Pdgfrb*, *Tgfbr2*) were markedly induced in carotid arteries from mice with CKD (Figure 2C).

Furthermore, the results from immunostaining showed that PDGFRA-positive cells colocalized with GLI1-positive (LacZ-positive) cells in artery adventitia (Figure 2D, left), and the remarkable induction of PDGFRA and LacZ was found in CKD mice, although the presence of autofluorescence of elastic laminae was also found (Figure 2D, right). Moreover, total RNA was extracted from adventitial layers of thoracic aortas of Ctl and CKD mice. Quantitative real-time reverse transcription PCR (RT-qPCR) analysis showed that CKD significantly stimulated the expression of *Pdgfra* and the components of *HH* signaling pathway (e.g., *Shh*, *Smo*, *Ptch1*, *Gli1*, *Gli2*) (Figure 2, E and F). These results from animal and patient AVFs confirm that CKD induced 2 events in adventitial cells: the increased expression of PDGFRA and the activation of HH signaling.

### Increased differentiation of myofibroblast-like cells from GMCs isolated from CKD mice.

**Figure 3:**
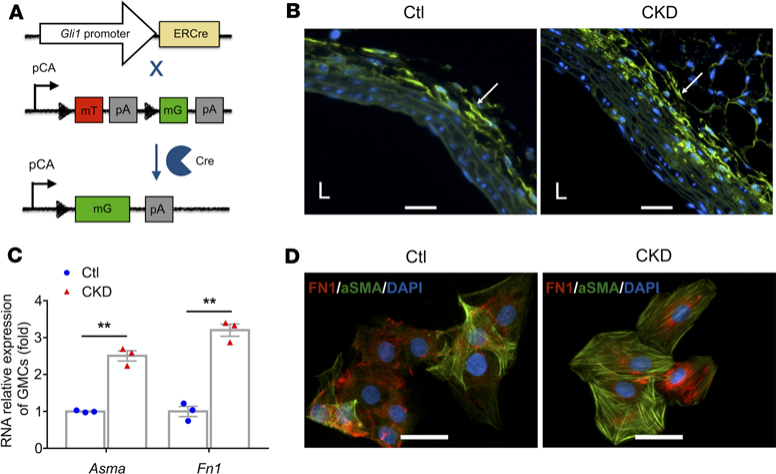
CKD stimulates GMC differentiation into myofibroblasts. (**A**) *GLI1^CreERt2^* mice were crossed with *mT/mG* reporter mice to generate *mT/mG/GLI1^CreERt2^* mice for labeling and tracking GLI1-positive cells. (**B**) GFP-positive cells were determined by immunostaining. More GFP-positive cells were found in the adventitia of carotid arteries from CKD versus sham Ctl mice. Scale bars: 100 μm. (**C**) Total RNAs were extracted from GMCs that were isolated from Ctl and CKD mice. CKD increased the mRNA levels of myofibroblast markers. The levels of mRNA expression are presented as folds of Ctl group after normalization to β-actin (*n* = 3). **P < 0.01 versus Ctls (*t* test). (**D**) GMCs isolated from Ctl and CKD mice differentiated into myofibroblasts. For immunofluorescent staining, GMCs were treated with TrueBlack Quencher reagent (40) (Biotium) for 15 minutes to quench GFP before incubating with fibronectin and α-SMA antibodies. Scale bars: 10 μm. CKD, chronic kidney disease; GMC, GLI1-positive mesenchymal stem cell; Ctl, control; α-SMA, α-smooth muscle actin.

To further analyze adventitia GLI1-positive cells, we crossed *Gli1-CreER^T2^* mice with membrane-targeted tandem dimer Tomato/membrane-targeted enhanced green fluorescent protein (mT/mG or GFP) reporter mice to generate mT/mG/*Gli1-CreER^T2^* mice. After Cre-mediated intrachromosomal recombination, the mT sequence was excised, allowing the promoter with a chicken actin promoter (pCA) to drive expression of mG. In these mice, GLI1-positive–expressing cells and their descendants were labeled with GFP after tamoxifen induction (Figure 3A). There were more GFP-positive cells in the adventitia of carotid arteries from CKD than in Ctl mice (Figure 3B).

Using FACS, the GFP-positive cells were isolated from adventitia of thoracic aortas of tamoxifen-treated mT/mG/ *Gli1-CreER^T2^* mice (Supplemental Figure 2A). Immunostaining results showed that these GFP-positive cells are stained positive for VIM, PDGFRA, PDGFRB, and the stem cell/progenitor markers, lymphocyte antigen 6 complex, locus A (LY6A, also called stem cell antigen 1 [SCA1]), and CD34 (Supplemental Figure 2B). These adventitia GMCs acquired multipotential differentiation characteristics when cultured in differentiation medium. They were capable of differentiating into adipocytes (Oil-Red O staining), osteoblasts (Von Kossa staining), and myofibroblasts (Supplemental Figure 2C). These results are consistent with a previous report (19).

Our previous study has shown that CKD increases collagen deposition in the adventitia and fibrosis in the vein and artery of the AVF (20). Notably, in this study, GMCs isolated from CKD mice had significantly higher levels of mRNAs of myofibroblast markers (Figure 3C) and showed higher capacities for differentiation into myofibroblasts when compared with cells isolated from sham Ctl mice (Figure 3D).

### PDGFRA was required for GMCs to differentiate into myofibroblasts.

**Figure 4:**
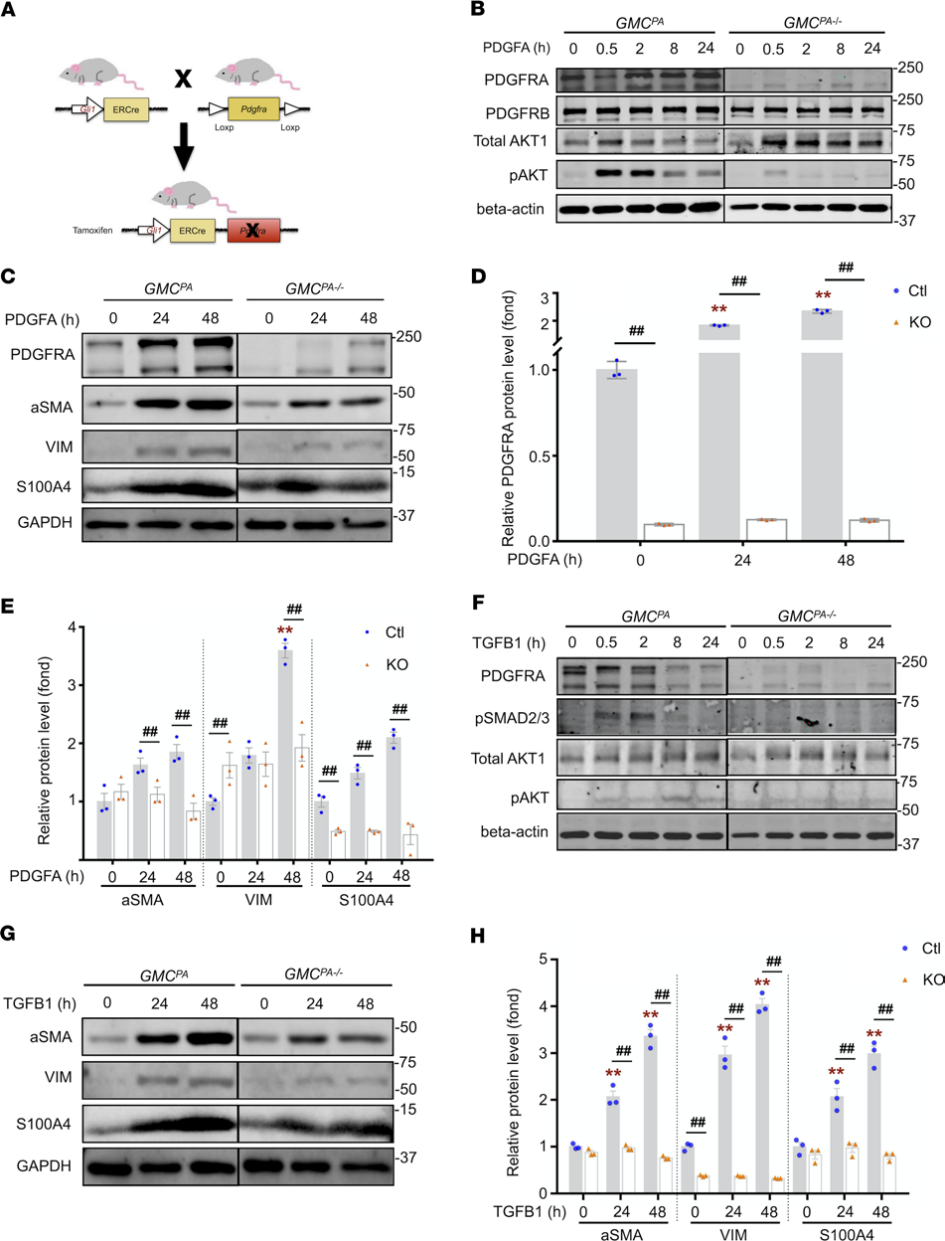
PDGFRA is required for GMCs to differentiate into myofibroblasts. (**A**) *GLI1^CreERt2^* mice were crossed with *PDGFRA^fl/fl^* mice to generate *GLI1^CreERt2^/PDGFRA^fl/fl^* mice (*GMC^PA–/–^* mice). (**B**) Representative Western blot (WB) of indicated proteins in both Ctl and *Pdgfra*-KO GMCs after treatment with PDGFA (10 ng/mL) for different times. (**C–E**) Representative images of WB showed that PDGFA time-dependently stimulated expression of PDGFRA and myofibroblast markers, S100A4, α-SMA, and VIM. In contrast, *Pdgfra* KO blocked PDGFA-induced promyogenic responses. Protein expression was normalized to GAPDH and expressed as fold change of Ctl GMCs (**D** and **E**). (**F**) Representative WB images of indicated signaling molecules in both Ctl and *Pdgfra*-KO GMCs after treatment with TGFB1 (2 ng/mL) for different times. Phosphorylation of SMAD2/3 and AKT1 present a parabolic change after TGFB1 treatment, whereas TGFB1-induced phosphorylation of SMAD2/3 and AKT were blocked in *Pdgfra*-KO GMCs. (**G** and **H**) Representative WB images of indicated myofibroblast markers in both Ctl and *Pdgfra*-KO GMCs after treatment with TGFB1 (2 ng/mL) for different times. Protein expression was normalized to GAPDH and expressed as fold change of Ctl GMCs (**H**). The lanes of gels were run on the same gel but were noncontiguous. Data are presented as mean ± SEM (*n* = 3). Two-way ANOVA was used for statistical analysis. **P < 0.01 versus Ctl 0 hour; ^##^*P* < 0.01 versus corresponding KO group. GMCs, GLI1-positive mesenchymal stem cells; Ctl, control; α-SMA, α-smooth muscle actin.

Because the expression of PDGFRA in GMCs is increased in CKD mice, we determined if PDGFRA regulates GMCs differentiation into myofibroblasts. We crossed *Gli1-CreER^T2^* mice with *Pdgfra^tm13Sor^* mice to generate GLI1^CreERt2^/*PDGFRA^fl/fl^* mice (*GMC^PA–/–^* mice) (Figure 4A). *Gli1-CreER^T2^* mice (*GMC^PA^*) served as Ctl. GMCs were isolated from *GMC^PA^* and *GMC^PA–/–^* mice after tamoxifen induction, and no PDGFRα expression was detected in GMCs isolated from *GMC^PA–/–^* mice (Supplemental Figure 3A).

Both PI3K and TGFB1 signaling pathways can regulate myofibroblast differentiation (21, 22), we determined if PDGFRA regulate these pathways during GMC differentiation. We found that PDGFA treatment increased the levels of PDGFRA and AKT1 phosphorylation (pAKT) in GMCs in a time-dependent manner, which was blocked by KO of *Pdgfra* (Figure 4B). In PDGFRA-positive cells, PDGFA time-dependently stimulated expression of myofibroblast markers, S100A4, α-SMA, and VIM, whereas KO of *Pdgfra* blocked PDGFA-induced myofibroblast differentiation (Figure 4, C–E). These results indicate that activation of PDGFRA in GMCs promotes GMC differentiation into myofibroblast.

Next, we determined if PDGFRA is involved in TGFB1-stimulated profibrogenic effects in GMCs. TGFB1 treatment stimulated SMAD2/3 phosphorylation in GMCs, which started at 0.5 hour and lasted for 2 hours. After 8 hours of TGFB1 treatment, there was increased pAKT and a decreased level of PDGFRA protein. KO of *Pdgfra* blocked TGFB1-induced phosphorylation of SMAD2/3 and AKT (Figure 4F). Furthermore, the TGFB1-induced expressions of myofibroblast markers, α-SMA, VIM, and S100A4, were abolished in GMCs lacking *Pdgfra* (Figure 4, G and H). These results indicate that the PDGFRA pathway is required for TGFB1-induced GMC differentiation into myofibroblasts.

### Internalization of PDGFRA in early endosome was required for TGFB1-induced GMC differentiation.

We have shown that PDGFRA is involved in TGFB1-induced GMC differentiation into myofibroblasts. Nevertheless, we also discovered that the PDGFRA level was decreased after TGFB1 treatment (Figure 4F). What is the physiological signification for the degradation of PDGFRA in TGFB1-induced myofibroblast activation? Most of the proteins are degraded by either the ubiquitin–proteasome system or the endosomal–lysosomal system. For the later pathway, molecules or ligands internalized from cell plasma membrane can follow endosome to lysosome for degradation, or they can be recycled to cell membrane in the endocytic cycle.

**Figure 5:**
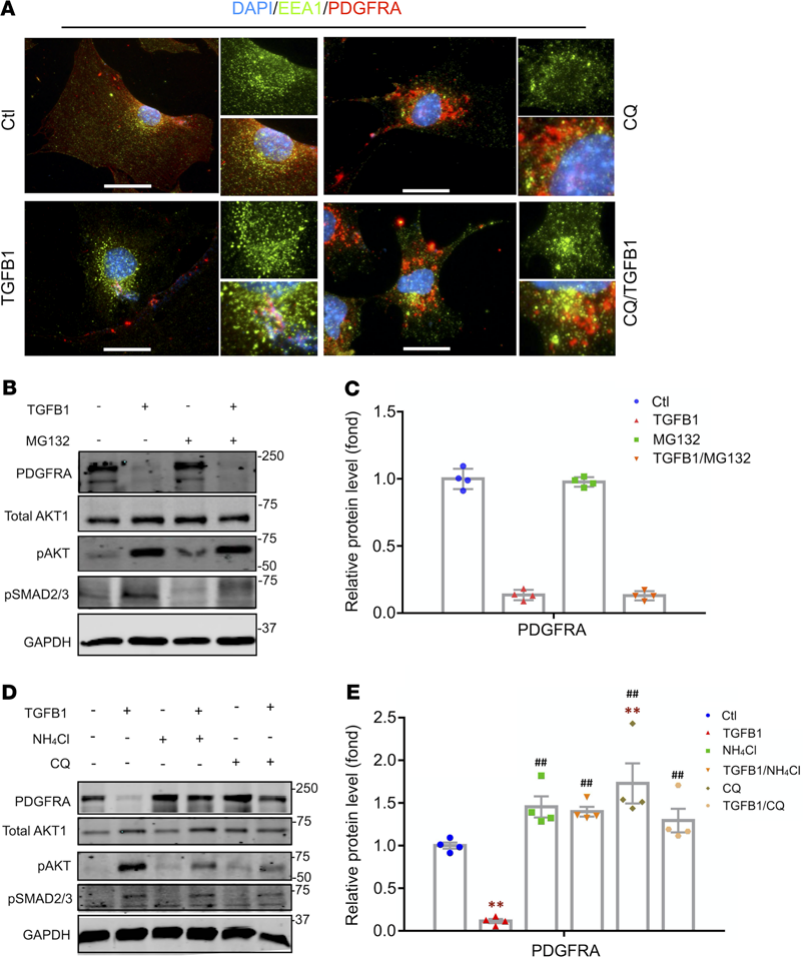
Internalization of PDGFRA in early endosome is required for TGFB1-induced GMC differentiation. (**A**) Representative images of the location of PDGFRA in the endosome (EEA1-positive) after different treatment. PDGFRA was accumulated during CQ treatment and was colocalized with EEA1 at the perinuclear area. Scale bars: 10 μm. (**B** and **C**) Western blot (WB) and densitometric analysis showing the changes of proteasome inhibitor (MG132, 10 μM) on the TGFB1-induced responses in GMCs. Protein expression of PDGFRA was normalized to GAPDH and expressed as fold change of Ctl GMCs (**C**). (**D** and **E**) WB and densitometric analysis showing the changes of endosome inhibitor (NH_4_Cl, 20 mM and CQ, 50 μM) on the TGFB1-induced PDGFRA degradation and activation of downstream signals in GMCs. Protein expression of PDGFRA was normalized to GAPDH and expressed as fold change of Ctl GMCs (**E**). Data are presented as mean ± SEM (*n* = 4). One-way ANOVA was used for statistical analysis. **P < 0.01 versus Ctl; ^##^*P* < 0.01 versus corresponding TGFB1-treated group. GMC, GLI1-positive mesenchymal stem cell; EEA1, early endosome antigen 1; Ctl, control.

To determine if the degradation of PDGFRA after TGFB1 treatment is processed in the endosome, we performed costaining of PDGFRA with early endosome antigen 1 (EEA1) (Figure 5A). In Ctl GMCs, PDGFRA localized mainly on plasma membrane with some in the cytoplasm, which was labeled with bright red staining. After TGFB1 treatment for 8 hours, EEA1 immunostaining became stronger and the red staining of PDGFRA had almost disappeared, suggesting that early endosome provides an important environment for cell signal interaction. In the presence of late endosome inhibitor chloroquine diphosphate (CQ), the granular PDGFRA signals gathered massively around the nucleus and colocalized with EEA1.

To further identify the PDGFRA degradation, GMCs were pretreated with the proteasome inhibitor (MG132) or with endosome/lysosome inhibitors (CQ or ammonium chloride [NH_4_Cl]) for 12 hours individually. We found that treatment with MG132 did not block TGFB1-induced degradation of PDGFRA, and it had no effects on TGFB1-induced phosphorylation of AKT and SMAD2/3 (Figure 5, B and C). In contrast, treatment with early endosome inhibitor, CQ or NH_4_Cl, blocked TGFB1-induced degradation of PDGFRA and suppressed TGFB1-induced phosphorylation of pAKT and SMAD2/3. (Figure 5, D and E).

These results indicate that the endosomal–lysosomal system participates in TGFB1-induced PDGFRA degradation and GMC differentiation into myofibroblasts. Internalization of PDGFRA is required for the profibrogenic activation of GMCs.

### HH signaling regulated PDGFRA expression and GMC differentiation.

**Figure 6:**
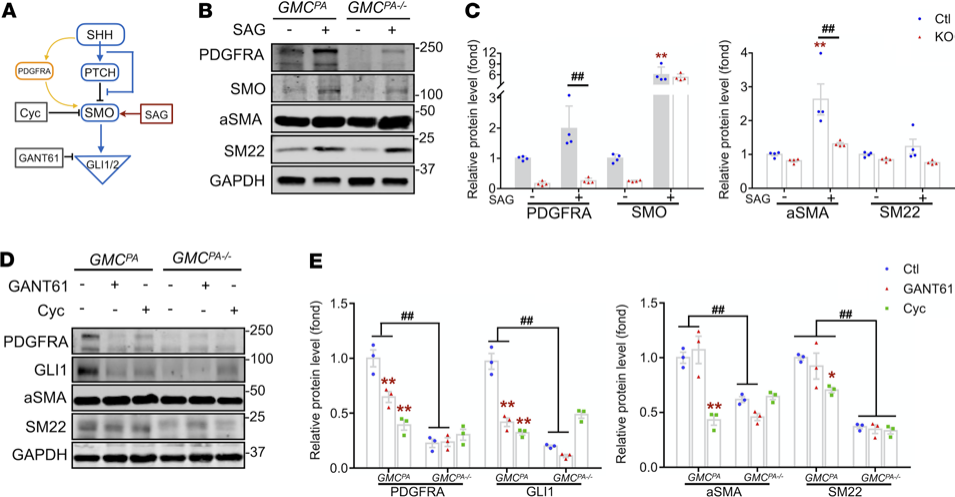
HH signaling regulates PDGFRA and the GMC differentiation. (**A**) Scheme of the targeting sites of the stimulator and inhibitors on HH signaling pathways. (**B** and **C**) Representative Western blot (WB) and densitometry graphs showing the effects of HH stimulator SAG (1 μM) on the level of PDGFRA, the activation of HH signaling, and the expression of myofibroblast markers in GMCs that were isolated from Ctl (*GMC^PA^*) and *Pdgfra*-KO (*GMC^PA–/–^*) mice. Protein expression was normalized to GAPDH and expressed as fold change of Ctl GMCs (**C**). Total replicates representing 3 independent experiments (*n* = 3). (**D** and **E**) Representative WB and densitometry graphs showing the effects of HH inhibitor cyclopamine (5 μM) or GANT61 (5 μM) on the level of PDGFRA, the activation of HH signaling, and the expression of myofibroblast markers in GMCs from Ctl (*GMC^PA^*) and *Pdgfra*-KO (*GMC^PA–/–^*) mice. Protein expression was normalized to GAPDH and expressed as fold change of Ctl GMCs (**E**). Total replicates representing 3 independent experiments (*n* = 3). Data are presented as mean ± SEM. Two-way ANOVA was used for statistical analysis. **P < 0.01 versus *GMC^PA^* Ctl group; ^##^*P* < 0.01 versus corresponding *GMC^PA–/–^* group. HH, hedgehog; GMCs, GLI1-positive mesenchymal stem cells; Ctl, control.

Our previous results (see above) reveal that activated HH signaling is associated with the increased expression of PDGFRA in adventitia of AVFs from patients with CKD or mice with CKD. We next determined if activation of HH signaling promotes the expression of PDGFRA in GMCs (Figure 6A). The smoothened agonist (SAG) is a chlorobenzothiophene compound that can activate HH signaling. SAG treatment for 24 hours increased expression of PDGFRA and SMO in GMCs. SAG also enhanced the expression of myofibroblast markers, α-SMA and transgelin (TAGLN, also called smooth muscle protein 22 [SM22]). KO of *Pdgfra* suppressed expression of SHH, α-SMA, and SM22 that were induced by SAG (Figure 6, B and C).

To inhibit HH signaling, we used cyclopamine (Cyc), a potent SMO inhibitor, and GANT61, an inhibitor of GLI1/2. Treatment with GANT61 or Cyc for 24 hours dramatically decreased the expression of PDGFRA and GLI1 in GMCs (Figure 6, D and E). Moreover, GANT61 or Cyc suppressed the expression of α-SMA and SM22. KO of *Pdgfra* overlaid the inhibitory effects. Thus, inhibition of HH signaling decreased PDGFRA expression and myofibroblast transformation of GMCs. These results demonstrate that HH signaling promoted the differentiation of myofibroblasts via regulating PDGFRA expression in GMCs.

### HH signaling promoted PDGFRA accumulation to enhance TGFB1-induced myofibroblast transformation.

Because our previous results (see above) reveal that PDGFRA internalization in early endosome is required for TGFB1-induced myofibroblast activation in GMCs, we next sought to determine if HH signaling pathway regulates these responses.

**Figure 7:**
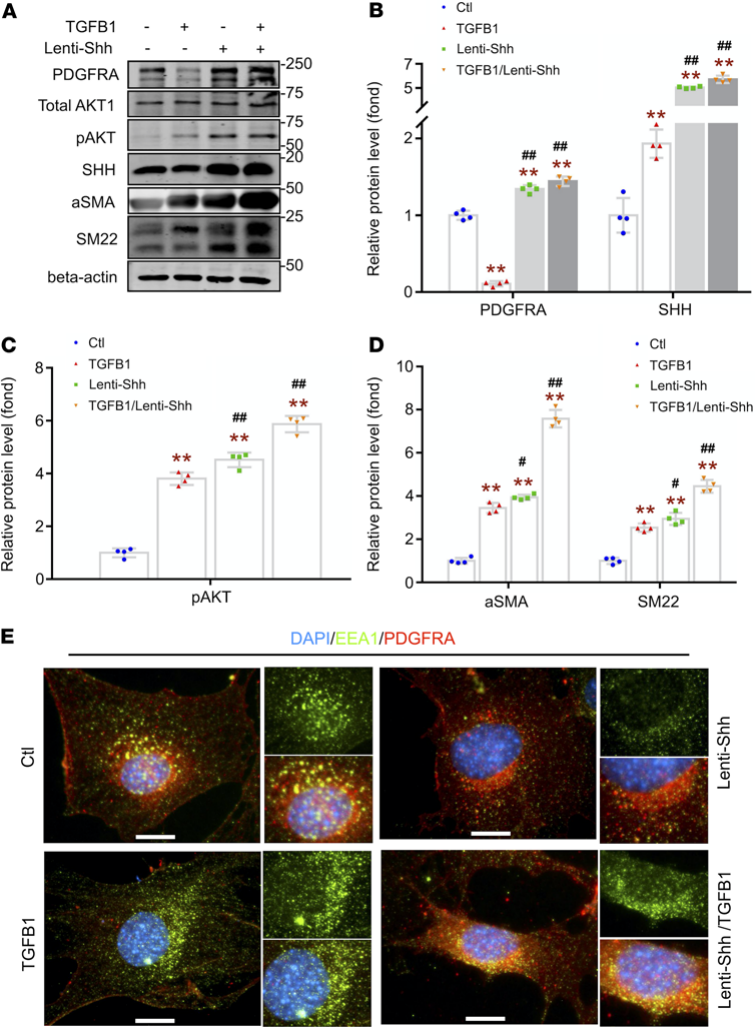
Hedgehog signaling promotes PDGFRA upregulation to enhance TGFB1-induced myofibroblast transformation of GMCs. (**A**–**D**) WB and densitometry graphs showing the effect of Lenti-Shh treatment on the TGFB1-induced PDGFRA degradation and expression of downstream signals in GMCs. (**B**–**D**) Protein expression was normalized to GAPDH and expressed as fold change of control GMCs. Total replicates represent 3 independent experiments (*n* = 3). (**E**) Representative images of the endosome and PDGFRA in GMCs treated with vector or Lenti-Shh. Scale bars: 10 μm. Data are presented as mean ± SEM. One-way ANOVA was used for statistical analysis. **P < 0.01 versus control; ^#^*P* < 0.05, ^##^*P* < 0.01 versus TGFB1-treated group.

We found that overexpression of SHH not only increased PDGFRA levels but also suppressed TGFB1-mediated PDGFRA degradation (Figure 7, A–D). Similar results were obtained when GMCs were treated with Lenti-GLI1 (Supplemental Figure 4). As expected, activating HH signaling stimulated phosphorylation of AKT and SMAD2/3, as well as the expression of the myofibroblast markers (α-SMA and SM22).

Because internalization of PDGFRA in endosomes is related to TGFB1-induced promyofibrotic differentiation of GMCs, we examined if HH signaling regulates the location of PDGFRA in GMCs (Figure 7E). PDGFRA localized on the cell membrane and distributed evenly in untreated GMCs. The PDGFRA almost disappeared after TGFB1 treatment. In contrast, SHH lentivirus treatment not only induced massive PDGFRA granules accumulating in membrane and cytoplasm but also suppressed TGFB1-induced PDGFRA degradation. Most of PDGFRA granules were colocalized with EEA1 at the perinuclear area (Figure 7E). These results suggest that HH signaling pathway can enhance TGFB1-induced myofibroblast differentiation of GMCs through regulating the synthesis, location, and activation of PDGFRA, which include the internalizing PDGFRA to early endosome.

### Pdgfra KO in GMCs suppressed CKD-induced neointima formation in AVFs.

**Figure 8:**
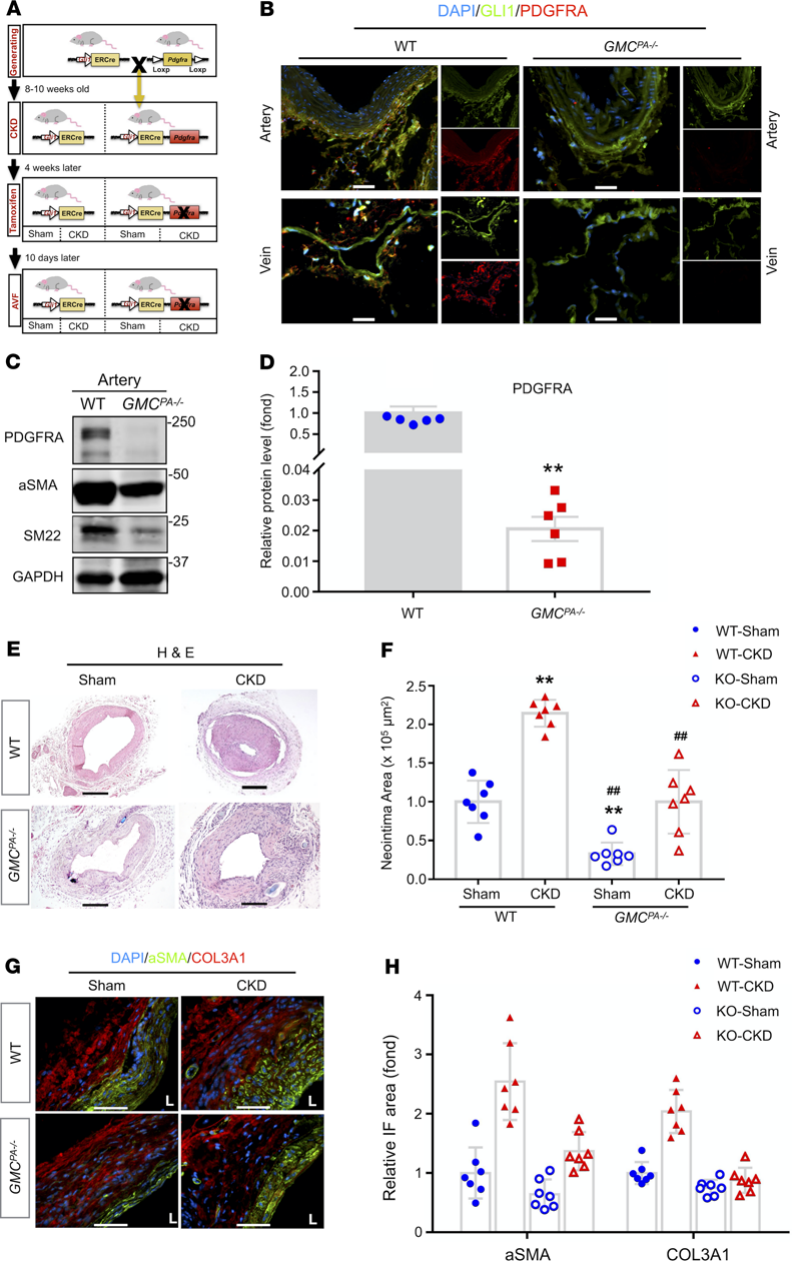
*Pdgfra* KO in GMCs suppresses CKD-induced neointima formation in AVFs. (**A**) The time course for creation of the CKD and AVF in WT (*Gli1-CreER^T2^*) and *GMC^PA–/–^* (*Gli1-CreER^T2^*/*PDGFRA^fl/fl^*) mice. (**B**) WT and *GMC^PA–/–^* mice were treated with tamoxifen or vehicle. The expression of PDGFRA and GLI1 in common carotid arteries and jugular veins was determined by immunostaining. Scale bars: 100 μm. (**C** and **D**) Representative Western blot (WB) and densitometric analysis of the expression of PDGFRA and myofibroblast markers in the arteries from WT and *GMC^PA–/–^* mice after tamoxifen treatment. Protein expression of PDGFRA was normalized to GAPDH and expressed as fold change of WT mice (**D**). Data were collected from 6 mice/group and presented as mean ± SEM. **P< 0.01 versus WT mice (*t* test). (**E**) Representative images of H&E staining of the AVFs created in WT and *GMC^PA–/–^* mice with or without CKD. Scale bars: 500 μm. (**F**) Quantification of the neointima area of AVFs created in WT and *GMC^PA–/–^* mice with or without CKD. Neointima area was expressed as fold change of WT/sham mouse. Data were collected from 7 mice/group and presented as mean ± SEM. **P < 0.01 versus WT/sham, ^##^*P* < 0.01 versus WT/CKD (1-way ANOVA). (**G** and **H**) Representative images of double immunofluorescent staining of α-SMA and COL3A1 of the AVFs created in WT and *GMC^PA–/–^* mice with or without CKD. Scale bars: 50 μm. Quantitative densitometry was normalized to DAPI area and expressed as fold change of WT/sham mouse (**H**). Data were collected from 7 mice/group and presented as mean ± SEM. **P < 0.01 versus WT/Sham, ^##^*P* < 0.01 versus WT/CKD (2-way ANOVA). GMCs, GLI1-positive mesenchymal stem cells; CKD, chronic kidney disease; AVFs, arteriovenous fistula; α-SMA, α-smooth muscle actin; COL3A1, collagen type III α 1.

To determine if the function of PDGFRA in adventitial GMCs in neointima formation, *Pdgfra*-specific KO mice were generated by crossing *Gli1-CreER^T2^* mice with *Pdgfra^tm13Sor^* mice. The generated *GMC^PA–/–^* mice and *GMC^PA^* WT mice were used for creation of the CKD and AVF (Figure 8A). The successful deletion of *Pdgfra* in GMCs of carotid arteries or vena cava was determined by immunofluorescence staining and Western blotting (Figure 8, B–D).

AVFs were collected 4 weeks after the AVF surgery. H&E staining revealed a smaller lumen and increased neointima area in AVFs placed in CKD versus sham WT mice. CKD-induced neointima formation in AVFs was suppressed in *GMC^PA–/–^* mice (Figure 8E). Quantification of the neointima areas indicated that AVFs created in *GMC^PA–/–^* mice had an approximately 40% decrease of neointima formation versus results from WT mice (Figure 8F).

There was accumulation of α-SMA–positive cells in the neointima and increased COL3A1 deposition in the adventitia of AVFs created in WT CKD mice. KO of *Pdgfra* in GMCs decreased the accumulation of α-SMA–positive and COL3A1-positive cells in AVFs (Figure 8G). The number of COL3A1-positive cells from AVFs of *Pdgfra*-KO mice were reduced by approximately 50% (Figure 8H). These data indicate that PDGFRA was involved in the CKD-accelerated neointima formation.

## Discussion

The vascular access is the lifeline for patients receiving hemodialysis, but successful maturation after surgery requires functional and structural adaptations to arterial blood flows (23, 24). The accumulation of VSMCs and myofibroblasts plus extracellular matrix deposition contribute to CKD-induced neointima formation in AVFs. The function of adventitial cells and the signaling pathways in regulating their activation and AVF remodeling remain largely unknown. We find that the increased expression of PDGFRA and activated HH signaling in adventitial GMCs stimulated differentiation of adventitial GMCs into myofibroblasts, leading to vascular stiffness and fibrosis. Based on results of the current study and previous reports (see above), we conclude that activated PDGFRA in MSCs triggers a negative remodeling of AVFs through the following processes. First, it stimulates adventitial fibrosis, leading to stiffening of the vein and artery. Fibrogenic vein and artery limit AVF outward expansion and induces inward remodeling. A recent report showed that collagen deposition in the vein is associated with AVF failure in patients with ESRD (25). Second, activation of PDGFRA in GMCs induces GMC differentiation into myofibroblasts or VSMCs, directly contributing to CKD-induced neointima formation. Consistently, Kramann reported that GLI1-positive MSCs are involved in neointima formation in an artery injury model (5). Third, our unpublished results showed that the adventitial GMCs interact with these VSMCs and promote VSMC activation and proliferation. These responses emphasize the function of PDGFRA in adventitial GMCs in regulating AVF remodeling and function.

The adventitia consists of a complex of interacting cells that are used to participate in growth, injury repair, and remodeling of blood vessel (6, 9). Neointimal hyperplasia thickens the vascular wall due to accumulation of VSMCs (26). The adventitial progenitor cells can differentiate into VSMCs and contribute to neointima formation (6). GMCs in adventitia can be genetically labeled in *Gli1-CreER^T2^* reporter mice. We and others find that adventitial GMCs express CD34 and SCA1 stem cell markers and can differentiate into adipocytes, osteoblasts, and myofibroblasts as well as VSMCs (4).

What mechanism regulates adventitial progenitor cell differentiation and incorporation into neointima? We find that HH signaling induces GMCs in the adventitia to proliferate and differentiate by regulating PDGFRA. HH signaling pathways that are activated during embryogenesis subsequently are silenced until recruited by tissue injury and regeneration (27, 28). For example, Dutzmann et al. (16) reported that activation of adventitial fibroblasts by SHH signaling stimulates fibroblast proliferation to expand the adventitial layer. Morrow and colleagues agree with this formulation but also conclude that HH signaling upregulates neointimal VSMC accumulation and increases NOTCH1 activation in vivo (29). Our results indicate that HH activation enhanced the expression of PDGFRA and promoted GMCs differentiation into myofibroblasts and VSMCs. Notably, we show that steady overexpression of SHH stabilized PDGFRA in early endosomes and sustained the activation of TGFB1 signaling pathway (Supplemental Figure 5). KO of *Pdgfra* in GMCs repressed GMC differentiation by compromising the activation of HH signaling.

Results from human AVFs showed that HH signaling was activated and then the expression of PDGFRA was increased in adventitial cells in AVFs. We also demonstrated increased expression of PDGFRA and activated HH signaling in our mouse AVF model. These cellular signaling pathways initiated GMC activation and differentiation, which lead to vascular stiffness and neointimal hyperplasia, finally resulting in the failure of AVF maturation.

The role of TGFB1 in neointima formation has been reported. Otasuka showed that overexpression of TGFB1 enhances neointima formation after artery injury (30). Others also prove that TGFB1 plays an important role in modulating repair of vascular injury, including restenosis, after balloon angioplasty (31). TGFB1 can trigger the differentiation of the adventitia progenitors (32, 33). In vivo, the overall effect of TGFB1 appears to be stimulation of neointimal hyperplasia (34, 35). We have shown that TGFB1 is stored in platelet and regulates remodeling of arteriovenous graft (36). In this study, we showed that the mRNA levels of *Tgfbr2* were markedly induced in carotid arteries from mice with CKD. TGFB1 treatment induced GMC differentiation. In AVF, adventitial progenitor cells responded to TGFB1, differentiated into myofibroblasts and VSMCs, and participated in the neointima formation.

Importantly, we found that PDGFRA pathway was required for TGFB1-induced GMC differentiation into myofibroblast by activating TGFB1/SMAD signaling in early endosome. PDGFRA is the modulator that controls TGFB1-induced GMC differentiation. Our results demonstrate that TGFB1 stimulated the internationalization of PDGFRA into early endosomes, where the PDGFRA can promote TGFB1 signaling. The absence of *Pdgfra* in GMCs inhibits TGFB1/SMAD signaling-induced myofibroblast differentiation. Consistently, Liu et al. reported that *Pdgfra* knockdown in hepatic cells can inhibit SMAD-dependent TGFB1 signaling (22). These findings demonstrate that PDGFRA interacts with TGFB1 signaling in promoting GMC differentiation and neointima formation.

In summary, we have documented that HH crosstalks with TGFB1-induced processing of PDGFRA in adventitial GMCs, which contribute to the CKD-induced neointima formation (Supplemental Figure 5). Internalization of PDGFRA to early endosome is required for TGFB1-induced myofibroblastic processing, which is regulated by HH signaling pathway. In a mouse model of CKD, we found that HH signaling stimulates PDGFRA expression and induces the neointima formation in AVFs. Knocking out *Pdgfra* in GMCs suppresses neointima formation of AVFs in CKD mice. Indeed, HH pathway inhibitors have been used in several preclinical and clinical studies and present the therapeutic activity in myeloproliferation disease. Our findings may translate into therapeutic approaches for reducing neointima formation and improvements in AVF maturation. Perhaps combinations PDGFRA inhibitors with inhibitors of the HH pathway could provide an avenue of targeting adventitia stem cell–derived fibrosis.

## Methods

### Reagents, antibodies, and virus.

Collagenase Type II (C6885) and animal experimental solution such as ketamine (K2753), xylazine (X1126), buprenorphine (B9275) were all purchased from MilliporeSigma. Elastase was obtained from Calbiochem (Merck KGaA). Penicillin/Streptomycin (Gibco, catalog 15140122), DMEM/F12 (Gibco, catalog 11320033), DMEM (Gibco, catalog 11995065), sheep anti–rat IgG (Dynabeads, catalog 11035), and FBS (Gibco, catalog 10100-147) were obtained from Invitrogen (Life Technologies). SAG (catalog 11914) and cyclopamine (catalog 11321) were obtained from Cayman Chemical. GANT61(G9048), MG132 (M8699), and NH_4_Cl (A9434) were obtained from MilliporeSigma, and chloroquine (catalog tlrl-chq) was obtained from InvivoGen. The Protein Assay Kit was obtained from Bio-Rad. Recombinant mouse SHH (catalog 461-SH-025), PDGFA (catalog 221-AA), and PDGFB (catalog 220-BB) proteins were purchased from R&D Systems. Recombinant human TGFB1 (eBioscience, catalog 14-8348) was purchased from Invitrogen. Monoclonal anti-mouse α-SMA (catalog A5228) and anti-SCA1 (AB4336) were obtained from MilliporeSigma. Shh (sc-365112), Gli2 (sc-271786), Smo (sc-166685), β-Galactosidase (anti-LacZ, sc-65670), COL3A1 (sc-271249), TGFB1 (sc-146), and SM22 (sc-53932) antibodies were obtained from Santa Cruz Biotechnology. Antibodies against pAKT (catalog 9275), AKT1 (catalog 9272), pSMAD2/3 (catalog 8828), PDGFRB (catalog 3169), CD34 (catalog 3569), VIM (catalog 5741), and GLI1 (catalog 2553) were obtained from Cell Signaling Biotechnology. Antibodies against GFP (ab290), S100A4 (ab197896), and fibronectin (ab2413) were obtained from Abcam. Goat anti-PDGFRA antibodies (AF1062) were purchased from R&D Systems. Antibody EEA1 (catalog 610456) was obtained from BD Biosciences. The fluorescent-700/800 secondary antibodies were obtained from Invitrogen. The BrdU Assay Kit (catalog 11296736001) was purchased from Roche. DAPI was obtained from Southern Biotech (catalog 0100-20).

The Lentivirus, Lenti-sg-Gli1 for KO *Gli1*, was constructed into CRISPR/Cas9 vector by following the protocol (24). The primers for mGli1-sgRNA were as follows: forward 5′-CACCGCTGGTGCTTGGCGCGGTCAC-3′, reverse 5′-AAACGTGACCGCGCCAAGCACCAGC-3′. To overexpress *Gli1* and *Shh*, the mouse *Shh* cDNA was cloned into FCGW lentivirus vector to construct Lenti-Shh. Primers for mouse *Shh* cDNA sequence were as follows: forward 5′-GCGGAATTCTCACTTGTCGTCGTCGTCTTTGTAGTCGCTGGACTTGACCGCCATT-3′, reverse 5′-GCGGGATCCATGCTGCTGCTGCTGGCCAGATGTTTTC-3′. The inserted length of *Shh* cDNA was 1.3Kb. Lenti-Gli1 vector (pLUT7 HA-GLI1, catalog 62970) and other packaging vectors such as pCMV-VSV-G (catalog 8454), pCMV delta R8.2 (catalog 12263), pCW-Cas9 (catalog 50661), lentiCRISPR v2 (catalog 52961), and lenti sgRNA(MS2)_zeo backbone (catalog 61427) for lentivirus were purchased from Addgene. All lentiviruses were packaged in HEK293T cells (Dharmacon, catalog HCL4517, General Electric Company).

### Human tissue collections.

Remnants of surgically excised circumferential cephalic and basilic veins were collected prospectively from patients with ESRD who underwent AVF surgery at Houston Methodist Hospital between July 2014 and June 2017. Parts of specimens were placed immediately into formalin and then embedded for H&E and immunostaining analysis. Parts of the specimens were placed immediately into liquid nitrogen for RNA extraction.

### Animals.

WT mice, *Gli1^tm2Alj^* mice (*Gli1^lz^*, JAX Stock, catalog 008211), *Gli1^tm3(re/ERT2)Alj/J^* mice (*Gli1-CreER^T2^*, JAX Stock, catalog 007913), *Pdgfra^tm13Sor^* mice (*PDGFRA^fl/fl^*, JAX Stock, catalog 006492) were purchased from Jackson Laboratory. Offspring were genotyped by Extract-N-Amp Tissue PCR Kit (XNAT2, MilliporeSigma) according to the protocol from the Jackson Laboratory. Mice heterozygous for the *LacZ* allele (*Gli1^lz+/–^*) were used as reporter lines. Mice were housed in a conventional animal facility with a 12-hour light/dark cycle. Mice between 1 and 6 months of age were used for all experiments. To induce gene knockout, tamoxifen (20 mg/mL in corn oil, T5648, MilliporeSigma) was given (i.p. 0.1 mL for 5 consecutive days).

### X-Gal staining.

Tissues of *Gli1^lz+/–^* were isolated and stained by using the X-Gal Staining Kit (catalog A10300K, Genlantis). Once color development had occurred, samples were fixed in 4% paraformaldehyde for 30 minutes, dehydrated through a sucrose gradient, and embedded in a sucrose-O.C.T.–embedding compound (catalog 4583, Tissue-Tek, Sakura Finetek) medium at a ratio of 1:3. Samples were cryosectioned at 8–10 μm on treated glass slides. FastRed Nuclear Counterstain (Vector Laboratories) was applied to the slides for 30 seconds to 1 minute. The slides were rinsed in double-distilled water and then dehydrated through an ethanol/xylene gradient and mounted.

### Mouse CKD model.

Mouse CKD model was created by subtotal nephrectomy in anesthetized mice as previously described (37, 38). Mice aged 8–10 weeks were used and divided into Ctl and CKD groups. After matching for body weights, subtotal nephrectomy was performed after anesthesia (ketamine at 125 mg/kg body wt. and xylazine at 6.4 mg/kg body wt.) in a 2-step surgery method. The left kidney was decapsulated to avoid ureter and adrenal damage and approximately three-quarters of it was removed. The right kidney was removed 1 week later. Slow-release buprenorphine (0.1–2.5 mg/kg body wt.) was given for pain relief before surgery. The BUN and relevant physiological factors, including the levels of hematocrit, were measured before AVF surgery by Comparative Pathology Laboratory Center in Baylor College of Medicine. The BUN levels between WT and *Pdgfra*-KO group have no significant differences. After 4 weeks of subtotal nephrectomy, AVFs were created in Ctl and CKD mice.

### Mouse AVF model.

Mouse AVFs were created as previously described (37). Briefly, mice were anesthetized, and the right internal jugular vein was isolated using a dissecting microscope (Leica MZ6). Its distal end was clamped and ligated, and the common carotid artery was clamped. An anastomosis was created using 12–0 nylon suture with an interrupted stitch. After unclamping, patency was confirmed visually. The mice were kept warm after surgery, and the analgesic (slow-release buprenorphine) was given 30 minutes before surgery. At 4 weeks after surgery, the mice were anesthetized by i.p. injection and euthanized by perfusing the left ventricle with PBS and 10% formalin for 10 minutes (to maintain the endothelium and morphology of the AVF). AVFs were harvested, and slides from the anastomosis to 1 mm from the venous anastomosis were harvested. Serial sections were prepared, and the slides at 0.2 mm near the venous anastomosis were used for morphometric and immunostaining analysis. All AVF figures in this article were taken from the venous anastomosis because the arterial anastomosis of AVFs has no significant neointima formation in this AVF mouse model (20). The area of neointima and media were defined as the regions between the lumen and the adventitia. The thickness of the vessel wall was determined by measuring the difference between the area of the lumen and the neointima, using the NIS-Elements BR 3.0 program (Nikon). Three cross-section slides were obtained by selecting the first of every 10 sections from each AVF. These slides were used to evaluate neointima formation.

### Primary vascular cell isolation.

Control in 1-month-old or CKD mice was used for primary cell isolation. After mice were euthanized, the apex of the heart was perfused with 10 mL of 1X sterile HBSS and then the thoracic aorta was dissected using a microscope. First, the fat tissue around the vessel was removed in ice-cold PBS. The adventitia layer was dissected from the aorta. The remaining aorta was cut open longitudinally, and the medium layer was prepared after removing the endothelial cells with a cotton swab. The dissected adventitial or medium layers were cut into small pieces individually, and then they were incubated with a digestion solution (5 mL HBSS, 5 mg Collagenase Type II, 10.8 μl [344U/mL] elastase, 50 μl Penicillin/Streptomycin) for 1 hour at 37℃ in a cell culture incubator. These 2 types of primary cells were washed individually and cultured with warmed DMEM/F12 media (1X DMEM/F12, 1% final Penicillin/Streptomycin, 20% final FBS). After centrifugation, the cells were resuspended with complete DMEM/F12 culture media and cultured. The medium was replaced every 3 days.

### FACS for GMCs.

WT and *Pdgfra*-KO primary adventitia cells were isolated from the thoracic aorta of *mT/mG/GLI1^CreERt2^* mice and *mT/mG/GLI1^CreERt2^/PDGFRA^fl/fl^* mice after tamoxifen treatment for 10 days. The trypsinized cells were suspended in sorting buffer (1X PBS, 1 mM EDTA, 25 mM HEPES, 1% FBS) by concentration of 7 x 10^6^ cells/mL and then transferred to 5 mL polystyrene round-bottom FACS tubes (BD Biosciences). FACS sorting was performed using the FACS Aria II cell sorter (BD Biosciences). The sorted cells were analyzed by using FlowJo software (version 9.6.2).

### GMC differentiation assays.

GMCs were plated in 48-well plate at a 60%–70% or 90%–100% confluence and culture medium was exchanged with Osteogenic or Adipogenesis Differentiation Kit medium (Thermo Fisher Scientific), respectively. After 21 days of cultivation, cells were stained according to manual protocols using Oil Red O Staining Kit (DIF001, MilliporeSigma) and Von Kossa Staining Kit (ab150687, abcam). GMCs were cultivated in myofibroblast differentiation medium (DMEM containing 20% FBS, 2% Penicillin Streptomycin, 10 ng/mL TGFB1, and 5 ng/mL PDGFB) for 14 days. Cell immunofluorescence staining for α-SMA and FN1 was performed. The Ctl cells were cultivated in the standard medium as mentioned above.

### Cell immunofluorescence staining.

Cells seeded on glass coverslips were rinsed with PBS and fixed in 4% Paraformaldehyde (catalog 158127, MilliporeSigma) for 10 minutes. Fixed-cells were processed with 0.1% Tween-20 and 0.1% Triton X-100 for 10 minutes at room temperature. After washing, the slides were blocked with 1% BSA (A2153, MilliporeSigma) in PBS for 30 minutes and then incubated with primary antibodies (prepared in 1% BSA solution) for 90 minutes at room temperature. After washing with PBS for 3 times, the coverslips were incubated with the fluorescent secondary antibody coupled for 30 minutes at room temperature. Specifically, GFP-positive GMCs were incubated in TrueBlack Quencher Reagent (catalog 23012, Biotium) for 15 minutes to quench GFP at the end of this step. The nuclei were counterstained with DAPI. Images were taken on a Nikon Eclipse 80i fluorescence microscope.

### IHC.

Tissues were fixed in 10% neutral-buffered formalin and paraffin-embedded. Briefly, after wax was removed, sections were incubated (30 minutes in 3% H_2_O_2_ in methanol at room temperature). They were subsequently washed with PBS after being heated in 10 mM citrate buffer (pH 6.0) for 20 minutes in a microwave. Tissue sections were stained with H&E. Histology was assessed in AVFs as previously described (39). Some sections were blocked with serum (Vector Laboratories) for 30 minutes before being incubated with the primary antibodies. Staining was performed according to the ABC Kit instructions (PK-6100, Vector Laboratories). Signals were visualized using a peroxidase substrate DAB Kit (SK-4100, Vector Laboratories), and photographs were recorded with staining intensity analyzed using the NIS-Elements BR 3.0 program (Nikon).

### Western blot.

Cells were washed with ice-cold PBS (6 mM Na_2_HPO_4_, 1 mM KH_2_PO_4_, pH 7.4, 140 mM NaCl, 3 mM KCl) and lysed in RIPA buffer (50 mM Tris pH 7.5, 150 mM NaCl, 1% NP40, 0.1% SDS, 0.5% Na Deoxycholate, 1 mM Orthovanadate, 5 mM NaF, 2.5 mM Na_4_P_2_O_7_) and cOmplete Protease Inhibitor Cocktail (catalog 12352200, Roche) for 20 minutes on ice. The cell lysates were centrifuged (8000*g*) for 10 minutes. Proteins (10–20 μg) were resolved on SDS-PAGE under reducing conditions on a 10% polyacrylamide gel. The Western blot system was established using the Bio-Rad Bis-Tris Gel system according to the manufacturer’s instructions. Primary antibodies were prepared in 5% BSA blocking buffer at a dilution of 1:1000 and incubated with the nitrocellulose membrane (Merck Millipore) at 4°C overnight, followed by washing and incubating with HRP-conjugated secondary antibodies for 1 hour at room temperature. The immunoblots were visualized by Odyssey V3.0 image scanning (LI-COR). The intensities of the bands were quantified using NIH ImageJ software, and the values were normalized to the β-actin or GAPDH values for each sample. And the relative protein levels were expressed as the fold change relative to the Ctls.

### RNA extraction and analysis.

Total RNAs were extracted with the TRIzol (catalog 15596-018, Invitrogen) according to manufacturer’s instructions. Total RNA was subjected to RT-qPCR analysis as previously described (29) by using a Reverse Transcription System Kit (catalog 170-8841, Bio-Rad) and a DNA Polymerase System Kit (catalog 12344-024, Invitrogen) according to manufacturers’ instructions. Primers were designed using Primer Express software (Applied Biosystems) and validated by testing PCR efficiency using standard curves (85% ≤ efficiency ≤ 115%). Gene expression was quantified using the comparative CT (threshold cycle) method on a StepOnePlus system (Applied Biosystems). And the relative mRNA levels were expressed as the fold change relative to the Ctls. GAPDH or β-actin was used as reference. Primers are listed in Supplemental Table 1.

### Statistics.

Data are presented as mean ± SEM. Comparisons between groups were analyzed using 2-tailed *t* test, 1-way ANOVA, or 2-way ANOVA as appropriate. A *P* value of less than 0.05 was considered to be statistically significant.

### Study approval.

All mouse experiments were approved by the Institutional Animal Care and Use Committee of Baylor College of Medicine and performed in accordance with NIH guidelines. Studies involving human AVF sections were approved by the Institutional Ethics Committee at the Houston Methodist Hospital.

## Author contributions

KS designed and carried out experiments, analyzed the results, and wrote the manuscript. YQ, QG, EKP, and JC carried out the experiments and analyzed the data. WEM, CC, and LC reviewed the manuscript. JC designed the experiments, interpreted the results, and wrote the manuscript.

## Supplementary Material

Supplemental data

## Version 1 10/01/2020

In-Press Preview

## Version 2 11/05/2020

Electronic publication

## Version 3 02/25/2021

Updated Methods section
