# Supplementary material for: PDGFRA in vascular adventitial MSCs promotes neointima formation in arteriovenous fistula in chronic kidney disease
Source: JCI Insight. 2020 Nov 5;5(21):e137298. doi: 10.1172/jci.insight.137298 (PMC7710276; doi:10.1172/jci.insight.137298)
Supplement: Supplemental data [file jciinsight-5-137298-s009.pdf]

ONLINE SUPPLEMENT

Non-standard Abbreviations and Acronyms	
**ACTA2**	actin alpha 2, smooth muscle (aSMA)
**AKT1**	AKT serine/threonine kinase 1
**AVF**	arteriovenous fistula
**CKD**	chronic kidney disease
**COL3A1**	collagen type III alpha 1
**CQ**	chloroquine
**Ctl**	control
**CVD**	cardiovascular risk
**Cyc**	cyclopamine
**EEA1**	early endosome antigen 1
**ESRD**	end-stage kidney disease
**FN1**	Fibronectin 1
**S100A4**	S100 calcium binding protein A4（FSP1）
**GFP**	green fluorescent protein
**GLI1**	GLI family zinc finger 1
**GMC**	adventitial GLI1^{+} mesenchymal stem cells
**KO**	knockout
**LacZ**	beta-galactosidase
**Ly6a**	lymphocyte antigen 6 complex, locus A (Sca1)
**MSCs**	mesenchymal stem cells

**NH_4Cl**	ammonium chloride
**PDGFA**	platelet derived growth factor subunit A
**PDGFB**	platelet derived growth factor subunit B
**PDGFRA**	platelet derived growth factor receptor alpha
**PDGFRB**	platelet derived growth factor receptor beta
**PTCH1**	patched 1
**RT-PCR**	reverse transcription polymerase chain reaction
**QPCR**	real-time polymerase chain reaction
**SAG**	smoothened agonist
**SCA1**	stem cells antigen 1
**SHH**	sonic hedgehog signaling molecule
**SM22**	smooth muscle protein 22 alpha
**SMO**	smoothened
**TAGLN**	transgelin (SM22)
**TGFB1**	transforming growth factor beta 1
**TGFBR2**	transforming growth factor beta receptor 2
**VEGFA**	vascular endothelial growth factor A
**VIM**	vimentin
**VSMC**	vascular smooth muscle cells
**WT**	wild type

Supplemental Table 1. Primer pairs used for qPCR

Gene	Sequence
Pdgfra	Fw 5’- TGGCATGATGGTCGATTCTA -3’<br>Rv 5’- CGCTGAGGTGGTAGAAGGAG -3’
Pdgfrb	Fw 5’-CACCTTCTCCAGTGTGCTGA -3’<br>Rv 5’-GGAGTCCATAGGGAGGAAGC -3’
Tgfbr2	Fw 5’- ACTGTCCACTTGCGACAACCAGA A -3’<br>Rv 5’- AGAAGCGGCATCTTCCAGAGTGAA -3’
Shh	Fw 5’- CTGGCCAGATGTTTTCTGGT -3’<br>Rv 5’- TAAAGGGGTCAGCTTTTTGG -3’
Smo	Fw 5’- GACCACTCCCATAAGGGCTA -3’<br>Rv 5’- GAAGAGGTTGGCCTAGTGGA -3’
Ptch1	Fw 5’- ATGCTCCTTTCCTCCTGAAACC -3’<br>Rv 5’- TGAACTGGGCAGCTATGAAGTC -3’
Gli1	Fw 5’- TCGACCTGCAAACCGTAATCC -3’<br>Rv 5’- TCCTAAAGAAGGGCTCATGGTA -3’

Gli2	Fw 5'- GGCAGGGGTATCCCACAGA -3'<br><br>Rv 5'- AGAAGGACCCATGTTGGAGTC- 3'

Supplemental figure legends

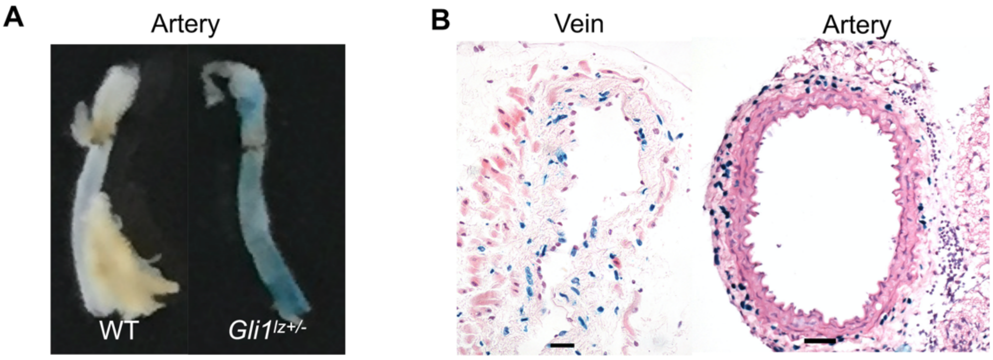

Song, K. et al. Supplemental Figure 1

Figure S1. GLI1 positive signals were restricted to the adventitial layers in *Gli1*$^{lz+/-}$ mice.

(**A**) Common carotid arteries from wide type and *Gli1*$^{lz+/-}$ mice were isolated. The arteries of *Gli1*$^{lz+/-}$ mice showed the blue color by X-gal staining.

(**B**) GLI1 positive signals were restricted to the adventitial layers of arteries and veins by X-gal staining. Scale bars are 100 μm.

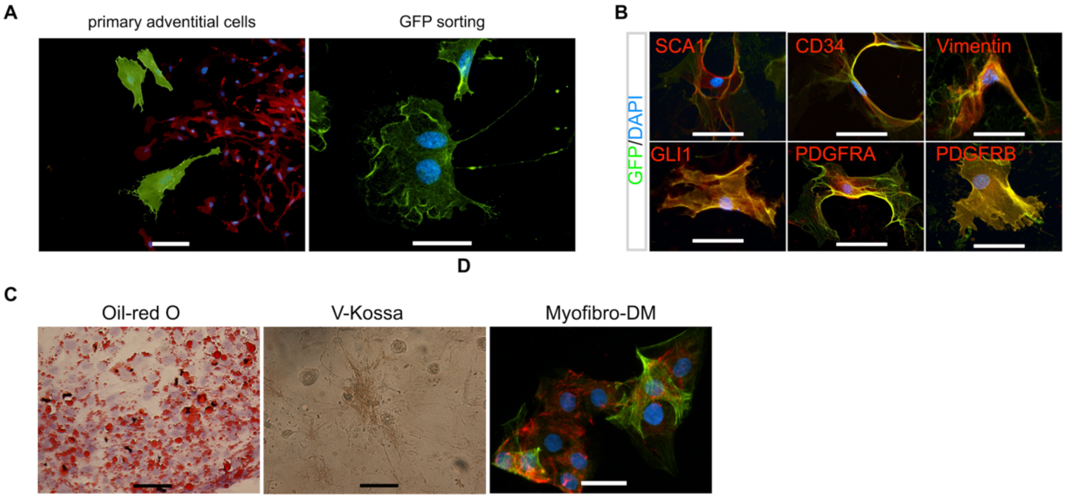

Song, K. et al. Supplemental Figure 2

Figure S2. GMCs isolated from the adventitia of aortas are MSC-like progenitors

(**A**) Primary adventitia cells were isolated from *mT/mG/GLI1*^{CreERt2} mice (left panel) and purified by sorting the GFP^{+} cells (right panel). Scale bars are 10 μm.

(**B**) Immunostaining revealed that GMCs were not only co-expressed with VIM and the stem cell/progenitor markers, SCA1 and CD34, but also expressed PDGFRA and PDGFRB. Scale bars are 10 μm.

(**C**) GMCs had the multipotential differentiation characteristics when cultured in differentiation medium. Scale bars are 50 μm for Oil-red O staining and Von Kossa staining, Scale bars are 10 μm for myofibroblasts differentiation.

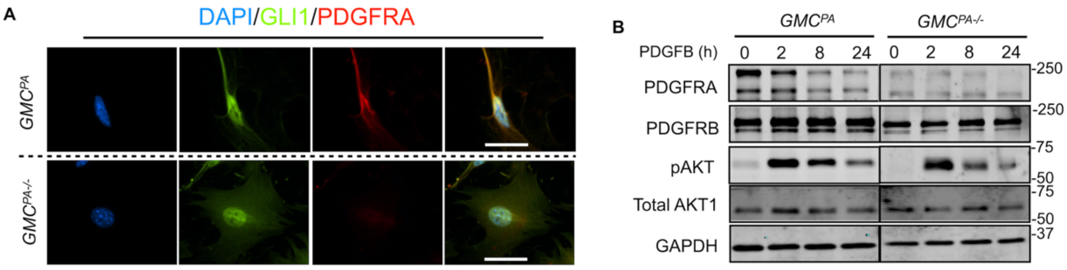

Song, K. et al. Supplemental Figure 3

Figure S3. GMCs were treated with PDGFB.

(**A**)GMCs were isolated from control (*GMC*^{PA}) and *GMC*$^{PA-/-}$ mice. After tamoxifen treatment, the expressions of PDGFRA and GLI1 were determined by immunostaining. Scale bars are 10 μm.

(**B**)Representative WB images of indicated proteins in both control and *Pdgfra* KO GMCs after treatment with PDGFB (10 ng/ml) for different time. The lanes of gels were run on the same gel but were noncontiguous.

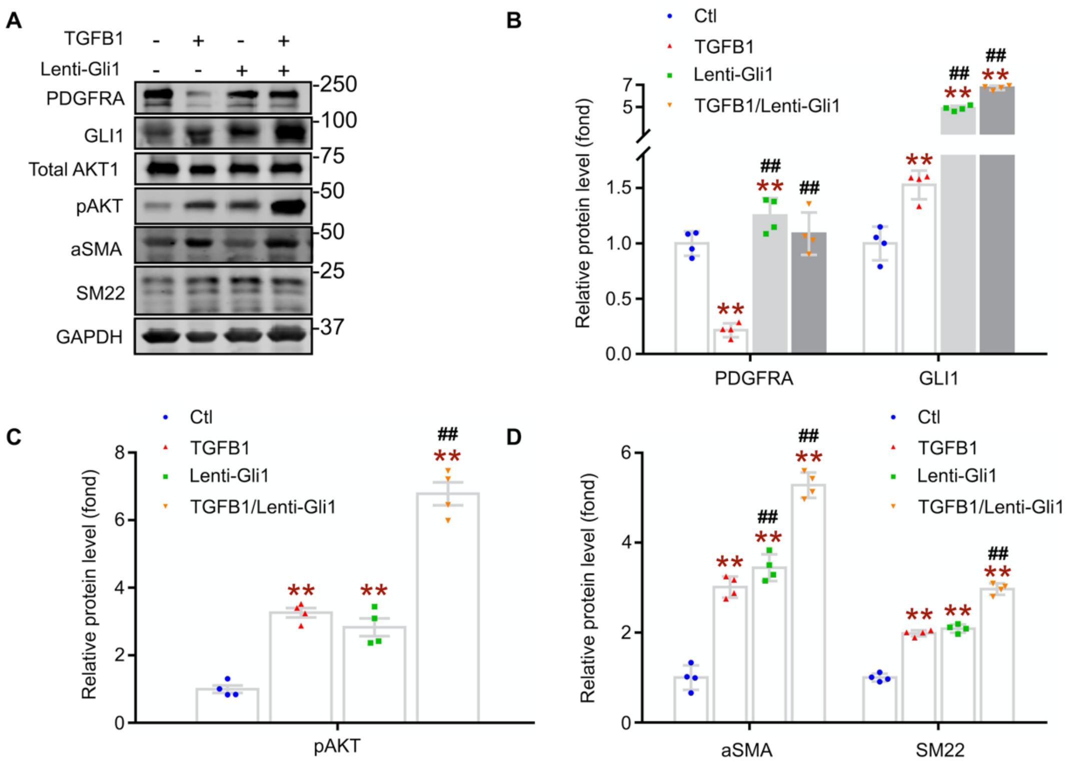

Song, K. et al. Supplemental Figure 4

Figure S4: Lenti-Gli1 promotes PDGFRA upregulation to enhance TGFB1-induced myofibroblast transformation of GMCs.

(**A-D**) WB and summary densitometry graph showing the changes of Lenti-Gli1 on the TGFB1-induced PDGFRA degradation and activation of downstream signals in GMCs. Protein expression was normalized to GAPDH and expressed as fold change of control GMCs(**B-D**). Total replicates representing 4 independent experiments (n = 4).

Data are presented as mean ± SEM. One-way ANOVA was used for statistical analysis. $^{**}P < 0.01$ *versus* control; $^{\#\#}P < 0.01$*versus* TGFB1-treated group.

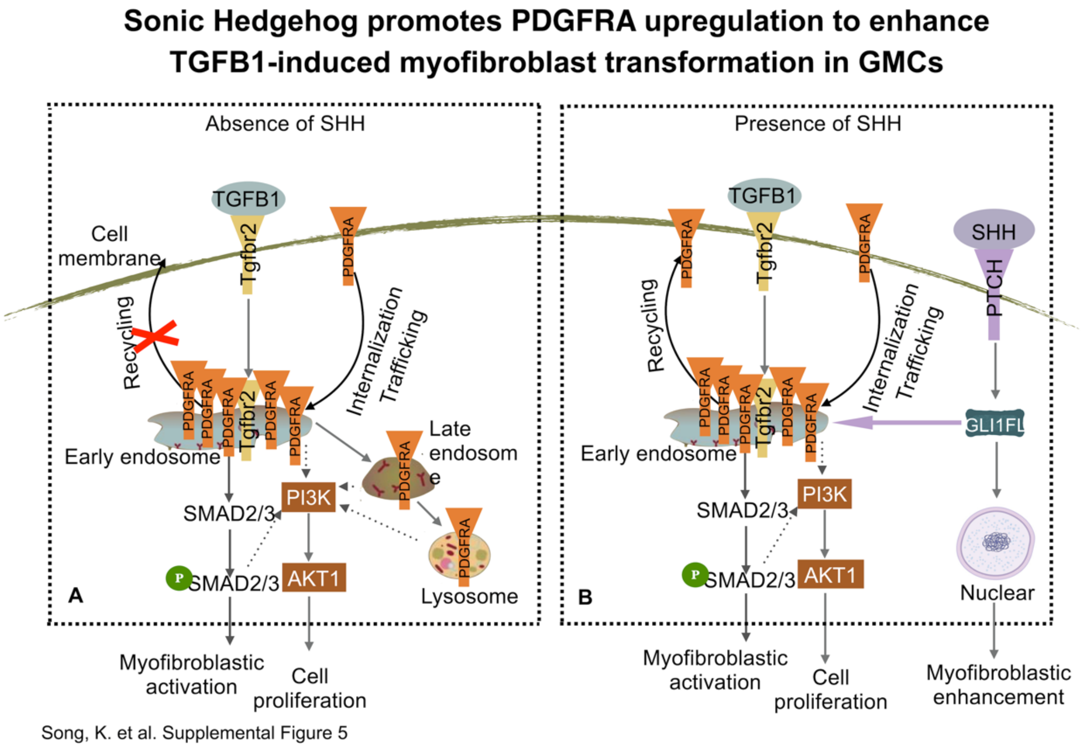

Figure S5. Sonic hedgehog promotes PDGFRA upregulation to enhance TGFB1-induced myofibroblast transformation in GMCs.

(**A**) In the absence of hedgehog ligands, TGFB1 treatment induces the degradation of PDGFRA through early endosome, leading to GMCs differentiation into myofibroblasts.

(**B**) In the presence of hedgehog ligands, the activated hedgehog signaling not only directly promotes GMCs differentiation, it also enhances PDGFRA accumulation in the early endosome, thus amplifying the TGFB1 induced GMCs activation and differentiation. These responses stimulate VSMC proliferation and extracellular matrix deposition, resulting in neointima formation in AVF in CKD.
